# *In vitro* gene silencing of independent phosphoglycerate mutase (iPGM) in the filarial parasite *Brugia malayi*

**DOI:** 10.1186/2049-9957-2-5

**Published:** 2013-03-25

**Authors:** Prashant Kumar Singh, Susheela Kushwaha, Shahab Mohd, Manisha Pathak, Shailja Misra-Bhattacharya

**Affiliations:** 1 Division of Parasitology, CSIR-Central Drug Research Institute, B.S. 10/1, Sector 10, Jankipuram Extension, Sitapur Road, Lucknow, UP, 226021, India

**Keywords:** siRNA, Lymphatic filariasis, Drug target, RNAi, Embryogenesis

## Abstract

**Background:**

The phosphoglycerate mutase (PGM) enzyme catalyzes the interconversion of 2- and 3-phosphoglycerate in the glycolytic /gluconeogenic pathways that are present in the majority of cellular organisms. They can be classified as cofactor-dependent PGM (dPGM) or cofactor-independent PGM (iPGM). Vertebrates, yeasts, and many bacteria have only dPGM, while higher plants, nematodes, archaea, and many other bacteria have only iPGM. A small number of bacteria, including *Escherichia coli* and certain archaea and protozoa, contain both forms. The silencing of *ipgm* in *Caenorhabditis elegans* (*C. elegans*) has demonstrated the importance of this enzyme in parasite viability and, therefore, its potential as an anthelmintic drug target. In this study, the role of the *Brugia malayi* (*B. malayi*) ipgm in parasite viability, microfilaria release, embryogenesis, and *in vivo* development of infective larvae post-gene silencing was explored by applying ribonucleic acid (RNA) interference studies.

**Results:**

The *in vitro ipgm* gene silencing by small interfering RNA (siRNA) leads to severe phenotypic deformities in the intrauterine developmental stages of female worms with a drastic reduction (~90%) in the motility of adult parasites and a significantly reduced (80%) release of microfilariae (mf) by female worms *in vitro*. Almost half of the *in vitro-*treated infective L3 displayed sluggish movement. The *in vivo* survival and development of siRNA-treated infective larvae (L3) was investigated in the peritoneal cavity of jirds where a ~45% reduction in adult worm establishment was observed.

**Conclusion:**

The findings clearly suggest that iPGM is essential for both larval and adult stages of *B. malayi* parasite and that it plays a pivotal role in female worm embryogenesis. The results thus validate the Bm-iPGM as a putative anti-filarial drug target.

## Multilingual abstract

Please see Additional file 1 for translations of the abstract into the six official working languages of the United Nations.

## Background

Lymphatic filariasis (LF) is a vector-borne helminth disease caused by slender nematodes, *Wuchereria bancrofti*, *Brugia malayi* (*B. malayi*), and *B. timori*. This incapacitating disease infects over 120 million people in 72 tropical and subtropical countries, while more than 1.39 billion people remain at the risk of infection [1]. The subclinical condition associated with patent infection may include acute manifestations, such as adenolymphangitis, acute dermatolymphangioadenitis, and tropical pulmonary eosinophilia that are rarely life threatening. However, chronic manifestations, such as lymphedema (elephantiasis) and hydrocele are debilitating, accounting for nearly five million disability-adjusted life years [2,3]. Current control measures for LF include annual doses of diethylcarbamazine or ivermectin alone, or in combination with albendazole, which principally targets microfilariae (mf) with little action on adult worms [4,5]. Target-based anthelmintic drug discovery is still at a nascent stage and only a few drug targets have been identified in filarial parasites utilizing this approach. The publication of the draft assembly of the *B. malayi* genome followed by an expansion in transcriptomic and genomic datasets has facilitated the identification of vital enzymes or proteins of filarial parasites that can be exploited as drug targets. This would further assist in the designing of potential anti-filarial compounds and in understanding of gene functions [6,7].

The gene silencing by ribonucleic acid (RNA) interference (RNAi) has been a landmark discovery in the area of biomedical research. RNAi has provided a functional genomics platform to unravel the functions of various genes and their associations with the disease state of the organism. ‘Target validation’ is an inherent aspect of the drug discovery process, which can be either accomplished by silencing the mRNA expression of a given gene *in situ*, or by using specific inhibitors against the target enzyme/protein to observe their deleterious effect on that organism. With a few exceptions, RNAi has not been utilized to its full extent in parasitic nematodes, including in filariids [8]. RNAi in *Caenorhabditis elegans* (*C. elegans*), a surrogate model for *B. malayi*, has been explored to identify the novel genes involved in nematode biology and comparative genomics studies have proposed a number of molecular targets for anti-parasitic drugs [9]. Another gene identified by RNAi in *C. elegans* is the 2, 3-bisphosphoglycerate independent phosphoglycerate mutase (iPGM). This enzyme's sequence and structure is completely different from the 2, 3-bisphosphoglycerate-dependent phosphoglycerate mutase (dPGM) found in mammals [10]. Both enzymes are responsible for the interconversion of 2-phosphoglycerate and 3-phosphoglycerate via different catalytic mechanisms. The down regulation of iPGM of *C. elegans* by RNAi resulted in embryonic and larval lethality [11]. Outcomes of RNAi experiments carried out on *C. elegans* do not always match those of parasitic helminths and, therefore, it becomes mandatory to carry out gene silencing in the target parasite itself [12]. The current investigation reports on the RNAi mediated *ipgm* gene silencing in adult and infective larval stages of human lymphatic filarial parasite, *B. malayi.* Using small size (small interfering) RNA (siRNA), the effect was observed on the parasite viability, the mf release, the adverse effect on the embryogenesis in the female worm, and further *in vivo* development of the treated infective larvae (L3) in the adult parasites in the peritoneal cavity of jirds.

## Methods

### Animals

Purpose-bred, parasite naïve, six- to eight-week old, male jirds (*Meriones unguiculatus*) were used in the study. Animals were maintained in proper housing conditions at an animal house facility at the Central Drug Research Institute (CDRI) in Lucknow, India, and fed a standard pellet diet and water *ad libitum*. The animals and the animal experimental procedures were approved by the Animal Ethics Committee of the Institute, duly constituted under the provisions of the Committee for the Purpose of Control and Supervision on Experiments on Animals (CPCSEA), Government of India. The study bears the approval no. 129/08/Para/IAEC/renew (84/09), dated 27.04.2009.

### Parasites and culture

The *B. malayi* infective larvae (L3) were recovered from the laboratory bred vector mosquito, *Aedes aegypti* and fed 9 ± 1 days earlier on the donor Mastomys (multimammate mouse-Mastomys *coucha*) [13]. The L3 were isolated from the gently-crushed mosquitoes using the Baermann technique, washed repeatedly in the Ringer’s solution, and counted. The six- to eight- week old male jirds were infected by inoculating 100–150 L3 into the peritoneal cavity of each animal. The adult parasites and the mf were recovered by washing the peritoneal cavity of the jirds after 120 to 180 days of intra-peritoneal infection. The mf were collected after passing the peritoneal lavage through a 5.0 μm membrane filter (Whatman, USA). The parasites were washed repeatedly in the fresh culture medium RPMI 1640 (Sigma, USA) containing 100 units/ml of penicillin and 100 μg/ml of streptomycin sulfate (Invitrogen, USA), and were transferred to an adequate volume of fresh medium, preheated to 37°C until they were ready to be used for RNAi experimentation. Undamaged, healthy, similar-sized, highly motile worms of both sexes, with females releasing almost near similar count of live mf, were selected for the RNAi experimentation.

### Design and synthesis of siRNA

The custom-designed and synthesized gene-specific siRNAs for Bm-iPGM used in this study were procured from Ambion, USA. The highest-ranking sense and anti-sense siRNA duplexes representing the best combination of activity and specificity were provided with a concentration of 40 nmoles as lyophilized powder. Stock solutions of 100 μM were prepared and stored at −20°C until use. The 5^′^-3^′^ sequences of the sense and antisense strands of siRNA were:

Sense (CCA UUG UGC UGA AAC AGA Att)

Antisense (UUC UGU UUC AGC ACA AUG Gaa)

The siRNA (#AM 4621, Ambion) completely unrelated to *B. malayi*, that does not target any gene product, was used as a negative control to determine off-target effects, if any. The negative control siRNA did not have any sequence similarity to that of mouse, rat, or human gene sequences, and have been pretested (Ambion) in cell-based screens and proven to have no significant effect on cell proliferation, viability, or morphology.

### Demonstration of *in vitro* siRNA uptake by parasites using fluorescence microscopy

The penetration of siRNA into *B. malayi* adult females, L3, and mf was observed after soaking the parasites in Cy3-labelled negative siRNA. Four female worms, 50 L3, and 100 mf were soaked in the medium containing 2 μM of siRNA separately for 24 hours at 37°C. All three parasite stages were washed repeatedly in PBS (pH 7.4), the fluorescence was visualized, and the parasites were photographed under a fluorescence microscope (Nikon, Japan) using a rhodamine filter set at an emission wavelength of 590 nm.

### siRNA treatment of *B. malayi* adult worms by the soaking method

The RNAi was carried out with the adult worms of both sexes by the soaking method. We used three groups in our experiments. The control group did not receive any siRNA treatment and the worms were kept in normal siRNA-free culture medium. The negative control group received the siRNA that was completely unrelated to *B. malayi* and the third experimental group received Bm-iPGM specific siRNA. The adult parasites (four female and two male worms) were taken in the midi GebaFlex tubes (cut off 5 kDa) containing 1 mM spermidine, 8U RNaseOUT (Invitrogen, USA), 5 μM of siRNA in 800 μl of RPMI medium fortified with 10% fetal bovine serum (Invitrogen, USA) medium, and six such tubes were kept in a beaker containing 300 ml of medium preheated to 37°C. The beaker with the tubes was placed in a CO_2_ incubator at 37°C and the tubes were retracted at various time periods. The first tube was removed after 12 hours of incubation and the adult worms from the tube were transferred to a fresh siRNA-free medium. The leftover medium in the tube was centrifuged at 800×g for two minutes, and the pellet was resuspended in another 50 μl medium for further observation under the compound microscope to assess the number and phenotype of the *in vitro* released mf. Of the four female worms, two were frozen in TRIzol reagent for later preparation of nucleic acid to measure mRNA expression of *Bm-ipgm*. The remaining two females and two males were transferred to the fresh pre-heated culture medium (37°C) for 30 minutes, and their motility was assessed individually and scored. The viability of each worm was subsequently checked by MTT reduction assay using the dye 3-(4,5 dimethylthiazol-2-yl)-2,5 diphenyltetrazolium bromide. The remaining three tubes were removed one by one after 24 hours, 36 hours and 48 hours, and were processed in the same way. The two leftover tubes were removed after 60 hours of incubation. Of the eight female worms obtained from these two tubes, two were frozen in the TRIzol reagent for their later use in PCR, while the remaining six females and four male worms were transferred to a fresh siRNA-free medium and were incubated for another 48 hours by replacing the medium with fresh normal medium every 24 hours. At the end of the experiment, i.e. 48 hours after shifting to the siRNA-free medium, two out of the six females were teased and their uterine contents were observed microscopically to determine the effect of gene silencing on embryogenesis by evaluating the relative proportion of various intrauterine progenies. The other two females were frozen in TRIzol reagent for PCR analysis, while the remaining two females and four male worms were checked for motility, viability, and *in vitro* mf release in the culture medium as discussed above. The experiment was repeated twice with the same number of worms under identical conditions and the data are expressed as the mean ± SD of the two experiments.

### The effect of gene silencing on the *in vitro* release of mf and phenotypic changes

The mf pellets, at various time points, were suspended in 50 μl of the PBS as mentioned above, and 10 μl of this suspension was used in triplicate for assessing the number of mf released *in vitro*. For observing the phenotypic changes, a thin smear of mf suspension was made on a glass slide, which was later fixed and stained with Giemsa and photographed (Nikon, Japan).

### The motility score and the viability of *B. malayi* adult worms

The viability of the male and the female worms was assessed by the mitochondrial reduction of 3-[4,5-dimethylthiazol-2-yl]-2,5-diphenyltetrazolium bromide (MTT, Sigma) to formazan, as described earlier [14]. The formation of the formazan was quantified spectrophotometrically at 530 nm in a multiplate reader (Tecan, Infinite M200, Switzerland), and the percent inhibition in MTT reduction by the treated parasite over that of control worms was assessed. The motility scoring of the adult worms was carried out as shown below and the percentage reduction in the motility of the worm over that of the control was evaluated. The worms displaying no movement even after transfer to the fresh siRNA-free medium were considered and scored as dead (see Table 1).

**Table 1:** Criterion for motility scoring of adult worms

**Motility of adult worms**						
**% Inhibition**	0	1 to 25	26 to 49	50 to 74	75 to 99	100
**Score**	5	4	3	2	1	Dead

### Measurement of the mRNA expression of *Bm-ipgm*

The loss of specific transcripts following the RNAi treatment was examined by real-time quantitative RT-PCR (qRT-PCR). The *B. malayi* β-tubulin gene (Bm-tub-1) was used as an endogenous control gene. For qRT-PCR, the primers were designed with the Beacon designer software (see Table 2). The frozen worms were homogenized in the TRIzol reagent and the RNA was extracted as described earlier [15]. The first strand cDNA was generated using the Super Script III first strand cDNA synthesis kit (Invitrogen, USA) using oligo (dT) 20 primers. The specific cDNA fragments were amplified by the real-time PCR (Roche, USA) using a SYBR premix. The PCR conditions for qRT-PCR were 95°C for 5 minutes, followed by 40 cycles of 95°C for 20 seconds, 56°C for 15 seconds, and 72°C for 30 seconds. The relative amount of target amplicon in each experiment was determined by the comparative ΔC_T_ method. The value of the control group (siRNA-free medium) was set to 100%, and the relative reduction in RNAi treated groups was calculated and expressed as percentage reductions.

**Table 2:** The PCR primers used for quantitative and RT-PCR analyses

**Target Gene**	**Primer name**	**Primers (5**^**′**^**-3**^**′**^**)**
Bm-ipgm	Bm-ipgm F	5^**′**^ GCT GAT AAG GTG ATT GAG 3^**′**^
	Bm-ipgm R	5^**′**^ CAG TTA CCA TCA GTA TGT AG 3^**′**^
Bm-tub-1	Bm-tub F	5 ACT TGG TGT CCG AAT ATC3^**′**^
	Bm-tub R	5^**′**^ACTCTTCCTGTTCAATGTAT3^**′**^

### The silencing of the *Bm-ipgm* gene in infective L3

Two hundred L3 were kept in each of the three wells of a 48-well plate containing 1 ml culture medium fortified with 1 mM spermidine and 8 U RNAse OUT. Of the three wells, one contained 2 μM gene specific siRNAs, another one contained non-target siRNA, while the third one was devoid of siRNA. The plate was incubated at 37°C in 5% CO_2_ for 24 to 48 hours, and the motility of the L3 was scored after 30 minutes of transfer to the fresh siRNA-free medium. The L3 that became totally immotile and did not display any sign of reversal in the motility after medium replenishment were considered to be dead. The L3 from individual wells were separately frozen in TRIzol reagent for analysis of Bm-iPGM transcript levels. Another set of experiments was conducted to observe the *in vivo* development of *in vitro* siRNA treated larvae in the naive jirds. The L3 were soaked in the gene-specific siRNA, non-target siRNA, and control media in the same manner as above for 24 hours, followed by washing in the siRNA-free medium, and 100 actively motile L3 each were subsequently inoculated in the peritoneal cavity of a 6-week old male jird. A total of three jirds/ experiment could be inoculated with 100 L3 each as only active and live larvae were used for infecting jirds. The jirds were euthanized after 120 days post-infection and the parasites were isolated by repeated washing of the peritoneal cavity. The parasites were counted, measured, and females were later teased individually in a drop of PBS to observe the intrauterine development.

### Statistical analysis

The data were analyzed using one-way and two-way (for grouped data) analysis of variance (ANOVA) with the help of statistical software PRISM 5.0. The individual comparisons following ANOVA were made using the Bonferroni method or the Newman-Keuls Multiple Comparison Test, wherever applicable. The criterion of evaluating statistical significance between the experimental and control groups was as follows: *p* value <0.05 was considered significant and marked as *, *p* <0.01 as highly significant and marked as **, and *p* <0.001 was very highly significant and marked as ***.

## Results

### The soaking method successfully delivered the siRNA

**Figure 1:**
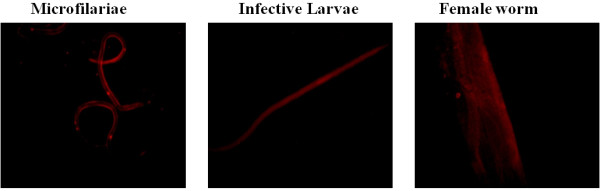
**Confirmation of penetration of siRNA to mf, L3, and adult female worms.** The Cy3-labeled negative control siRNA was taken up by all three stages of parasite *in vitro* after soaking for 24 hours at 2 μM concentration. The fluorescence was visualized using a Nikon fluorescence microscope using the rhodamine filter set at emission 590 nm.

All three parasite stages were incubated *in vitro* in the presence of 2 μM concentration of Cy3-labeled negative control siRNA to ensure the proper uptake of siRNA by the soaking method. The siRNA uptake occurred successfully in all three stages of *B. malayi* at 24 hours of soaking (see Figure 1). Fluorescence could be clearly detected at 24 hours in the hypodermis, intestine, and uterus of the female worms, while in the L3 and mf, siRNA could be visualized throughout the body of the filarial parasites.

### The silencing of the *B. malayi ipgm* gene impairs the adult *B. malayi* viability

The siRNA-mediated silencing of the *Bm-ipgm* gene by the soaking method resulted in the reduced motility of both male and female worms as compared to that of the control worms, which were exposed either to the normal, medium, or negative siRNA (see Table 3). Male worms were more sluggish in appearance as compared to the female worms in the experimental groups. The siRNA-treated parasites showed reduced viability in MTT reduction tests, however, both the male and female worms remained alive until the end of the experiment (60 hours), and also after 48 hours of transfer to the siRNA-free medium (total 108 hours). The treated worms demonstrated a continuous decrease in their motility with the increasing duration of incubation in specific siRNA, and the motility data correlated well with the inhibition in MTT reduction. Maximum inhibitions in MTT reductions in treated female and male worms were observed to be 75.9% and 89.4%, respectively. After 36 hours of soaking in the medium-containing target siRNA, both sexes of adult parasites demonstrated ≥50% inhibition in MTT reduction that exhibits a significant adverse effect on parasite viability [16].

**Table 3:** **Effect of *Bm-ipgm *gene silencing on adult male and female *B. malayi***

	**Motility score (female worms)**			**% Inhibition in MTT reduction**		Motility score *(male worms)			**% Inhibition in MTT reduction**	
***Time*( *hr *)**	***Control***	***Negative siRNA***	***Gene specific siRNA***	***Negative siRNA***	***Gene specific siRNA***	***Control***	***Negative siRNA***	***Gene specific siRNA***	***Negative siRNA***	***Gene specific siRNA***
**12**	5	5	4	2.25 ± 3.3	29.9 ± 5.4	5	5	4	2.5 ± 1.3	26.8 ± 4.5
**24**	5	5	3	4.5 ± 1.2	40.2 ± 6.2	5	5	3	4.5 ± 1.3	38.8 ± 6.5
**36**	4	5	3	5.0 ± 2.3	55.4 ± 6.3	4	4	2	4.5 ± 2.6	64.4 ± 5.4
**48**	4	4	2	6.2 ± 2.8	74.4 ± 3.4	4	4	1	6 ± 1.8	80.2 ± 6.8
**108**	4	4	2	6.26 ± 2.3	75.9 ± 4.7	4	4	1	5.25 ± 2.6	89.4 ± 5.3

**Key:** *0%: 5; 1 to 25%: 4; 26 to 49%: 3; 50 to 74%: 2; 75-99%: 1; 100%: Dead.

### The *Bm-ipgm* silencing impairs the release of mf by the female worms, mf motility and their phenotype *in vitro*

**Figure 2:**
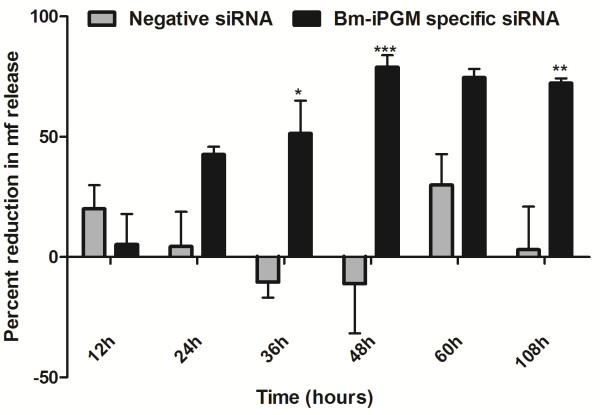
**The *in vitro *siRNA treatment of adult female *B. malayi*leads to a reduction in microfilaria release.** Tubes containing six adult worms each were treated with 5 μM siRNA for different time periods (12, 24, 36, 48, 60 hours). After 60 hours of treatment, six females were cultured for a further two days in fresh siRNA-free normal culture medium (108 hours). The percent reduction in the mf release was analyzed in the Bm-iPGM-specific siRNA-treated and negative control group over that of the control media group. There was a significant reduction in the release of mf by Bm-iPGM siRNA-treated worms starting from 12 hours and continuing until 108 hours. The criterion for statistical significance between the results was carried out by ANNOVA and was expressed in the form of *p* value that was considered significant and marked as *p* <0.05 *, *p* <0.01 as highly significant **, and *p* <0.001 *** as very highly significant. Data represent mean ± SE of percent reduction in mf counts from two independent experiments.

**Figure 3:**
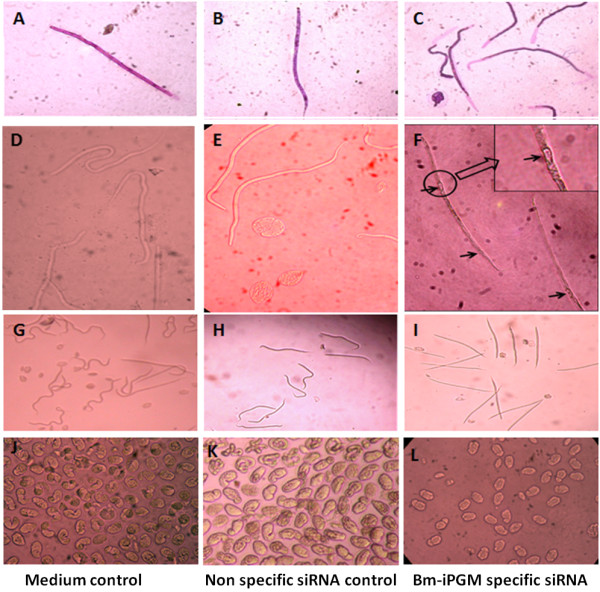
**The *Bm-ipgm *specific gene silencing causes several developmental defects in the mf and the eggs.** The Giemsa-stained mf from the control group (**A**), siRNA-free negative control group (**B**), and the Bm-iPGM specific siRNA-treated group (**C**) exhibited stretched mf sheaths. The mf from the control groups (**D** and **E**) had normal phenotypes as compared to Bm-iPGM-specific siRNA treatment that led to variable phenotypes in the mf including degenerating internal contents containing several vacuoles (**F**, arrows). The mf from the control groups were alive (**G**, **H**), while those from the specific siRNA-treated group were dead (**I**). The eggs from the control groups (**J**, **K**) were normal, while those from the iPGM specific siRNA (**L**) were degenerated, showing increased spaces between the eggshell and the embryo.

The mf released by the controls and the siRNA-treated female *B. malayi* in culture medium were counted. A considerable drop was noticed in the number of mf released by the *Bm-ipgm* specific siRNA-treated female parasites within 24 hours of incubation, which decreased further up to ~80% (*p* <0.001) at 48 hours, indicating a potent adverse effect of gene silencing on mf release. This effect remained unchanged even after transferring these worms to a siRNA-free medium. No such pattern was observed in the non-specific siRNA-treated control worms (see Figure 2). In addition, a complete loss of motility was also witnessed in ~80% of the released mf (these appeared dead) within 24 hours of exposure and partial paralysis was observed in the remaining 20% mf (these seldom even had slight movement of the anterior or posterior portion tips). The phenotypic deformities in the mf were conspicuous by contraction of the body at one end, leaving most of the mf sheath empty. Besides this, a few small and large vacuoles were also visible inside the non-motile mf in the *Bm-ipgm* specific siRNA-treated group (see Figure 3).

### The *Bm-ipgm* gene silencing had profound adverse effects on female worm embryogenesis

**Figure 4:**
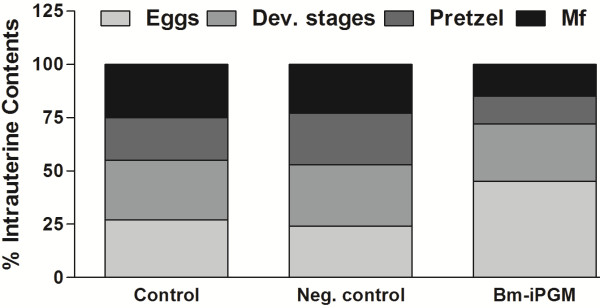
**The effect of siRNA on *B. malayi *female worm embryogenesis.** The embryograms of the female worms exposed to the culture medium, the negative siRNA, and the Bm-iPGM-specific siRNA (5 μM) were examined after 108 hours of incubation. The data are expressed as percentages of eggs, early developing stages, pretzel, and mf.

At the end of the experiment (108 hours after initiation of RNAi treatment), the female worms were collected for preparing the embryogram (see Figure 4). The relative proportion of various progenies at different stages of development that displayed good proportions of the intrauterine contents in the form of degenerated eggs (~40%) was noted, and the complete morphogenesis seemed to have failed. The remaining stages consisted of eggs, early embryos, pretzel and, full-grown mf. The silencing of the *Bm-ipgm* gene caused the abnormality in the phenotypes of the eggs and the mf. All the developing stages appeared normal in the culture medium devoid of the target siRNA, or the non-target probe (see Figure 3).

### Soaking of the adult *B. malayi* in the *Bm-ipgm* siRNA-containing medium leads to the loss of the Bm-iPGM transcript

**Figure 5:**
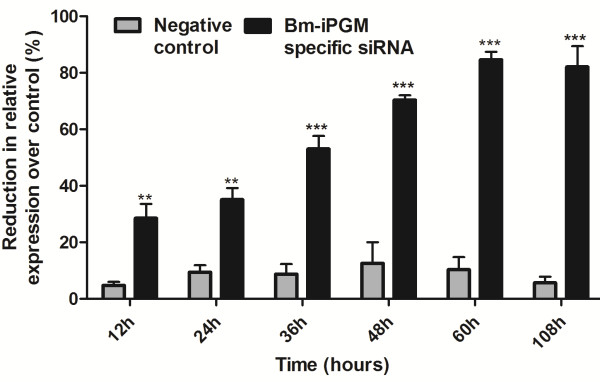
**The percent reduction in gene transcript level in *Bm-ipgm *specific siRNA treated worms.** Two female worms at different time intervals (12, 24, 36, 48, 60 hours) and 48 hours after transfer to normal siRNA-free culture medium were removed and analyzed for differences in *Bm-ipgm* gene-specific transcript levels by qRT-PCR. The relative amounts of iPGM amplicon were determined by using the comparative ΔCT method and normalizing against the endogenous control gene (Bm-tub-1). The median value of the control group was set to 100% and the reduction in expression in the treated groups was calculated as a percentage of the control. The criterion for statistical significance between the results was carried out by ANNOVA and was expressed in the form of *p* value that was considered significant and marked as *p* <0.05 *, *p* <0.01 as highly significant **, and *p* <0.001 *** as very highly significant. Data represent mean ± SE of percent reduction in mf counts from two independent experiments.

The qRT-PCR results showed that the *Bm*-*ipgm* gene silencing resulted in the transcript reduction relative to the expression of the β-tubulin control gene within 12 hours, which continued further until 60 hours. This reduction in the gene expression profile of the siRNA-soaked worms was irreversible even after 48 hours of transfer to the siRNA-free medium (see Figure 5). The control worms revealed the presence of the Bm-iPGM transcript at all time points of the study. The reduction in the Bm-iPGM specific transcript level was highly significant (*p* <0.001) when compared with the negative control, which had negligible (5-8%) reduction. The data have been represented as the percentage reduction in the gene expression normalized using β-tubulin (Bm-tub-1) transcript as the housekeeping gene control. The reduction in the transcript levels correlated well with the reduction in the release of mf from the target specific siRNA-treated female worms, phenotypic abnormalities, and with the complete loss of motility of the released mf, as well as with adverse effects on the intrauterine development.

### The *Bm-ipgm gene* silencing in the infective L3 reduces their motility and impairs their further development in jirds

**Figure 6:**
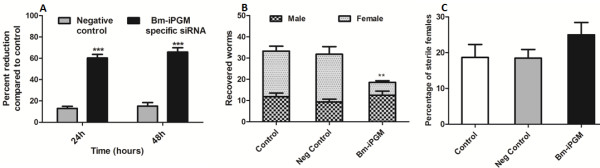
**Effect of siRNA on *Bm-ipgm *gene transcription in the L3 stage and the production of adult worms.** (**A**) siRNA treatment of infective larvae (L3) resulted in a significant loss of the Bm-iPGM transcript level after 24 and 48 hours of incubation. (**B**) The number of recovered male and female worms from the control, negative control, and *Bm-ipgm*-specific treated L3 implanted jirds. The *Bm-ipgm*-specific siRNA-treated L3 exhibited 44.36% reduction in the adult worm establishment in the peritoneal cavity of naive male jird, which was significant (*p* <0.01) over control. (**C**) The percentage of sterile female worms recovered from control, negative control, and *Bm-ipgm*-specific treated L3 implanted jirds. A significant number of female worms recovered from the siRNA-treated group displayed defective embryogenesis. The recovered females were teased in a drop of PBS on glass slide and the condition of intrauterine contents was analyzed microscopically (Nikon). The percentage of sterile female worms was calculated with respect to the total female worms recovered and analyzed. The criterion for statistical significance between the results was carried out by ANNOVA and was expressed in the form of *p* value that was considered significant and marked as *p* <0.05 *, *p* <0.01 as highly significant **, and *p* <0.001 *** as very highly significant. The data represent the mean ± SE of percent reduction in mf counts from the two independent experiments.

*In vitro* silencing of the *Bm*-*ipgm* gene in the L3 brought about a reduction in the motility of almost half the cultured L3 at 48 hours in contrast to the control group, where 10-12% of the L3 became sluggish. Under normal culture conditions in the normal medium, around 25% of the L3 became non-motile and were considered dead. A similar effect was also observed in the target siRNA-treated group (see Table 4) demonstrating no noticeable adverse effects of siRNA on larval viability. However, the gene silencing had profound adverse effects on the L3 motility leading to sluggishness but not amounting to death within 48 hours of observation. There was ~65% reduction in the Bm-iPGM transcript level as revealed by the qRT-PCR of these L3 at 24 and 48 hours of observation (see Figure 6A). The surviving active and motile L3 from all three groups from the different batches were separately inoculated into the peritoneal cavity of the naïve jirds to observe further *in vivo* development of these L3s. There was a 44.36% reduced recovery rate (*p* <0.01) of adult parasites when jirds were euthanized on day 120, in comparison to those recovered from control jirds (see Figure 6B). A proportion of recovered female worms (24.9 ± 6.8%) from experimental jirds contained degenerated intrauterine contents (see Figure 6C). An interesting observation was that the relative establishment of female worms was very low in the jirds infected with the target gene specific siRNA-treated L3 in contrast to the female worm recovery from the two control groups. The sizes of the adult parasites were also marginally shorter than those isolated from the control jirds (see Table 5).

**Table 4:** The effect of Bm-iPGM siRNA treatment on the morphology of infective larvae (L3)

**Infective Larvae condition**	**Control**		**Negative control**		**Bm-iPGM specific siRNA**	
	**24 h**	**48 h**	**24 h**	**48 h**	**24 h**	**48 h**
***Active (%)***	82.5 ± 4.9	58.5 ± 12.0	82.5 ± 7.7	57.5 ± 9.1	49 ± 2.12	23.5 ± 9.1
***Sluggish (%)***	10.0 ± 2.8	17.5 ± 3.5	12.5 ± 3.5	17.0 ± 5.6	37.0 ± 2.12	50.5 ± 3.5
Dead* (%)	7.5 ± 2.2	24.0 ± 8.4	10.0 ± 2.8	25.5 ± 3.5	14.0 ± 4.24	26.0 ± 12.7

*: These were the non-motile L3 where no reversal in motility was noticed even after transfer to siRNA-free medium.

**Table 5:** Measurement of worm length recovered from jirds infected with siRNA-treated and untreated infective larvae

**Groups**	**Worm length (cm ± S.D.)**	
	***Female***	***Male***
***Control***	3.47 ± 0.57	1.67 ± 0.34
***Negative control***	3.48 ± 0.65	1.75 ± 0.25
**Bm-iPGM*specific siRNA***	2.60 ± 0.70	1.58 ± 0.29

## Discussion

Phosphoglycerate mutases are the enzymes that catalyze the interconversion of 2- and 3-phosphoglycerate in the glycolytic/gluconeogenic pathways that are present in most cellular organisms. High-throughput RNAi studies performed in *C. elegans* have identified several genes essential for parasite viability including the cofactor-independent phosphoglycerate mutase (iPGM) [17-20]. The iPGM is present in almost all nematodes, bacteria, and trypanosomes with the exception of some bacteria, such as *E. coli*, and certain archaea and protozoa that contain both the iPGM and dPGM forms. [21-27]. Because of its absence from the vertebrates and its key role in pathogen development, this enzyme shows great potential as a possible drug/vaccine candidate. In this study, attempts were made to investigate the functional role of the *B. malayi ipgm* gene in parasite viability, mf release, embryogenesis, and *in vivo* infective L3 development by RNAi mediated silencing using two important life stages of *B. malayi*, the L3 and adult worms.

The RNAi was discovered accidentally in *C. elegans*[28,29] and has been tried with parasitic nematodes with variable and limited success. *Nippostrongylus brasiliensis* was the first nematode for which RNAi effects were reported using double-stranded RNAs (dsRNAs) [30] and since then, RNAi has been reported from various other nematodes, such as *Haemonchus contortus*[31-33], *Ascaris suum*[34-36], *Trichostrongylus colubriformis*[35,37], *O. volvulus*[38,39], *Ostertagia ostertagi*[37], and *B. malayi*[40]. Recently, *in vivo* silencing has also been demonstrated in *Aedes aegypti,* the mosquito vector of *B. malayi*[41]. We have recently reported on the use of 19 bp siRNA to successfully silence *B. malayi* RNA helicase by both soaking and electroporation methods [42].

Selection of siRNAs appropriate for a given sequence are of prime importance and are mainly based on structural attributes, such as optimal length, concentration, the requirement of a 3’ dinucleotide overhang, and a low G/C content [43-46]. Initially, we used 21 bp siRNA for soaking the mf, L3, or adult worms at 2 μM concentration of Cy3-labeled, non-specific siRNA, which showed a successful penetration of siRNA in all three parasite stages. However, 5 μM of siRNA was used for soaking adult parasites in our experiments, as this concentration was effective enough to bring down the silencing effects in our previous studies. For the gene silencing in the L3, a lower concentration (2 μM) of siRNA was used as L3s demonstrated better absorption of siRNA at this lower concentration. The low concentrations and small-sized dsRNA/siRNA have also been found to produce more effective gene silencing effects as opposed to high concentrations that induce stress in the parasites. The small-sized siRNA reduces the innate immune interferon response and produces negligible non-target effects [42,47-49]. The single siRNA was computationally designed and custom synthesized by Ambion to target Bm-iPGM.

Several studies have reported on off-target effects of gene silencing and, therefore, use of more than one siRNA has been recommended to confer effective gene silencing. To confirm that the observed effects were gene specific, a negative control completely unrelated to *B. malayi* was used in this study. However, the use of another siRNA specific to Bm-iPGM would have given our results even more credibility. In previous studies, we have used a single siRNA to target specific genes of interest and were substantially successful in gene silencing without observing any off-target effects [38,46]. The soaking method was used, as described earlier [40,42,50], with substantial modifications. Worms were kept in different tubes so that each tube could be taken out at any time point without touching the remaining worms in the other tubes. This was done to avoid any mechanical damage, stress, or contamination to the remaining parasites requiring longer incubation. The loss of gene transcript level started within 12 hours of soaking in the Bm-iPGM specific siRNA, and was apparent until the end of the experiment, not showing any reversal even after 48 hours of further transfer of worms to the siRNA-free medium. The loss of gene function slowed down the motility of both the male and female parasites. The percent inhibition in parasite motility correlated very well with the MTT assay, which is a quantitative assay and has been widely used for evaluation of *in vitro* anti-filarial activity of compounds against *B. malayi* worms. There was a specific knockdown of the Bm-iPGM transcript along with a range of deleterious effects on the phenotypes, however, additional studies could not be performed to detect the degree of protein knockdown. This was due to the limited number of experimental and control worms, and these were utilized for assessing other parameters thought to be more important for the study.

The measurement of the mf release in the culture is a robust method to ensure the ill effects of gene silencing on the female parasites. The profound decrease in the number of mf released by the specific siRNA-treated female worms indicated the deleterious effects on worm function. The released mf revealed phenotypic deformities in the form of a contracted body at one end, leaving a long sheath at the other end, as well as internal vacuole formation with subsequent loss of motility, amounting to death. The relative proportion of various progenies demonstrated failure of complete morphogenesis. The number of free mf and pretzel stages within the uteri of the female worms showed considerable reduction with increased proportion of early egg stages showing impairment in the transformation of the divided eggs into the pretzel stages. This might have been brought about by the insufficient production of energy needed for germ-cell differentiation since iPGM is a crucial enzyme of the parasite glycolytic pathway. It has been shown that mitochondrial content is regulated during transition from different parasitic stages and different stages have different energy requirements [51]. iPGM is involved in fundamental metabolic pathway and has been shown in *C. elegans* to be expressed throughout the worm in all the developmental stages, being abundant in the cells with higher metabolic rates, such as contracting body wall muscles, nerve ring, and the intestinal cells.

The disruption of the *C. elegans* iPGM by RNAi brought about variable defects, including embryonic and larval lethality [11]. Almost half of the *in vitro* siRNA- treated L3 became sluggish, while a further 25% remained totally immotile even after transfer to normal culture media and were thus considered ‘dead’. Even though, the gene silencing effects were conspicuous on the motility and viability of L3, it could not lead to their death. The possible reason for infective L3 survival could be the abundant storage of the iPGM enzyme in the worm body, or the slow and sustained depletion of the iPGM transcript of up to 90%, but not 100%. A single concentration of siRNA was used in the current study due to its high cost, however, increasing the duration of the siRNA treatment or the use of different concentrations of siRNA may have possibly shown dose dependent effects on *B. malayi*.

A successful RNAi experiment depends on various factors, including culture conditions, route/mode of siRNA delivery, the gene targeted, its site, the level of expression in the parasite, and the parasite itself. This indicates differences between nematodes in the uptake and the spread of dsRNA/siRNA that could have been the reason for the variation in the results obtained with different parasites. Silencing of the iPGM in *C. elegans* had some differing outcomes that could be because of the differences in the RNAi methodology used by us and Zhang et al., 2004 [10]. The latter have used dsRNA instead of siRNA, which was used in our study. The dsRNA/siRNA design could inform variable efficiencies of different dsRNAs/siRNAs or individual transcript sensitivities within the individual species, and this could have been one of the reasons for the differences observed. However, in *C. elegans*, the embryonic lethality started within 18 to 24 hours, reaching a maximum of 94% at 50 to 60 hours post injection of the dsRNA.

The *in vivo* survival and the development of siRNA-treated L3 was further investigated by peritoneal inoculation in the naive jirds and euthanizing of the animals on day 120 to observe the establishment of these larvae into the adult parasites. The *in vivo* results demonstrated the inability of a good proportion of the treated infective L3s to reach adult stage. Not only this, but the established adult worms had profound retardation in their lengths over those who recovered from the control groups. In addition, there was an unusually low recovery rate of female worms, which was less than half of the recovered males. This may be due to differential *Bm-ipgm* expression patterns in male and female worms. In one study, it has been shown that approximately 4% of genes are associated with glycolysis and gluconeogenesis in male worms as compared to only 1% in female worms [52]. Thus, silencing of the ipgm might have had a more fatal effect on the carbohydrate metabolism in female worms resulting in their reduced recovery. The control groups had almost twice the number of female worms than male worms, which is quite usual in *B. malayi*-infected animals. The recovered female worms from the experimental group also displayed defective embryogenesis. These *in vivo* studies confirm the role of iPGM in nematode metabolism, growth, and fecundity. The adverse effects of iPGM gene silencing on growth have been demonstrated earlier in a variety of plants and human parasites. The double iPGM mutants in *Arabidopsis thaliana* and iPGM antisense potato plants showed severely-retarded plant growth that correlated with the decreased concentrations of phosphoenolpyruvate [53,54]. The iPGM is required for the normal growth of *Trypanosoma brucei* procyclic [26] and the blood stream form [55], and has been proposed as a drug target in spore-forming *Bacillus* species and tomato pathogen, *Pseudomonas syringae*[56,57].

The present findings clearly demonstrate the vital role of iPGM, a metabolic enzyme in parasite growth, and validates Bm-iPGM as a possible anti-filarial drug target.

## Conclusion

The identification and validation of anti-filarial drug target/s is of extreme importance as there is currently no adulticidal anti-filarial drug. RNAi has been widely used to validate and identify such drug targets, and PGM is one such enzyme found in two forms: vertebrates contain the dPGM form, while the iPGM is present in various parasites, including filariids. The siRNA treatment led to several phenotypic deformities in the intrauterine stages of treated female worms. A drastic reduction (~90%) was noticed in adult parasite motility along with a significant reduction (80%) in the *in vitro* release of mf from female worms. Almost half the *in vitro*-treated L3 displayed sluggish movement. The important aspect of the study was the observation of *in vivo* survival and development of the siRNA-treated L3 in jirds that showed ~45% reduction in the adult worm establishment. The information obtained from this study clearly indicates the necessity of Bm-iPGM in the life cycle of *B*. *malayi*, and emphasizes its major role in female worm embryogenesis. The study validates Bm-iPGM as a potent anti-filarial drug target that can be utilized to design its novel inhibitors. Further structural and docking studies are underway to facilitate the design and synthesis of such Bm-iPGM inhibitors that would facilitate anti-filarial drug discovery programs.

## Competing interests

The authors declare that they have no competing interests.

## Authors’ contributions

SMB conceived the study, analyzed the data, and drafted the manuscript. PKS and SK initiated the study, performed all the molecular and RNAi experiments, analyzed the data, and drafted the manuscript. MS and MP contributed to the experimentation. All the authors contributed to the writing of the manuscript and approved the submitted version.

## Supplementary Material

Additional file 1 Multilingual abstracts in the six official working languages of the United Nations. Click here for file
