# Supplementary material for: In vitro gene silencing of independent phosphoglycerate mutase (iPGM) in the filarial parasite Brugia malayi
Source: Infect Dis Poverty. 2013 Mar 25;2:5. doi: 10.1186/2049-9957-2-5 (PMC3707094; doi:10.1186/2049-9957-2-5)
Supplement: Additional file 1 — Multilingual abstracts in the six official working languages of the United Nations. [file 2049-9957-2-5-S1.pdf]

Translation of the abstrat into the six offical working languages of the United Nations

تجربة مختبرية لإيقاف عمل جين إنزيم فُسْفُوغِليسَرات موتاز المستقل (**IPGM**) في الطفيل الفيلاري بروجية ملاوي

براشانت كومار سينغ - سوشيلا كوشيواها - موهد. شهاب - مانيشا باثاك - شايلجا ميسرا- باختاريا Prashant Kumar Singh, Susheela Kushwaha, Mohd. Shahab, Manisha Pathak, and Shailja Misra-Bhattacharya

ملخص

نبذة تاريخية: يقوم إنزيم فُسْفُوغِليسَرات موتاز (PGM) بتحفيز التحورالكامل فُسْفُوغِليسَرات 2 و3 في ممرات إنزيم حالٌّ للسُّكَّر/ استحداث السكر جيني المنشأ الموجود في معظم الكائنات الخلوية. حيث يمكن تصنيفها على إنها عوامل مساعدة معتمدة على PGM (dPGM) أو على إنها عوامل مساعدة مستقلة عن PGM (IPGM). و يوجد في الفقاريات والخميرة والعديد من البكتريا النوع المساعد المعتمد فقط DPGM بينما يوجد في النباتات الأعلى و الخيطيات و البدئيات وأنواع البكتريا الأخرى النوع المساعد المستقل فقط iPGM إلا أنه يوجد في عدد صغير من أنواع البكتريا وتتضمن عصويات القولون وبعض البدئيات والبروتوزا يوجد كل من النوعين وقد أظهرت عملية إيقاف *Ipgm* في الربداء الرشيقة *C. elegans* (*Caenorhabditis elegans*) أهمية هذا الإنزيم في استمرار حياة الطفيليات ولذا يمكن أن يستهدف كعقار طارد للديدان. وقد كان دور بروجيه ملاوي (ب.ملاوي) في هذه الدراسة دراسة ipgm في حياة الطفيليات و إطلاق الميكروفيلاريا والتَخَلُّق وفي التأثيرات الحيوية لتطور اليرقانات الغير فعالة قبل الإيقاف الجيني للإنزيم وقد تم استكشاف هذا عن طريق تطبيق دراسات التدخل باستخدام حَمْضُ الريبونوكلييك RNA

النتائج: وقد أدى الإيقاف المختبري لإنزيم *IPGM*عن طريق التدخل الطفيف ب RNA (siRNA) إلى تشوهات ذات نمط ظاهرى في مراحل التطور الرحمي لإناث الديدان مع إنخفاض حاد بنسبة 90%) في قدرة الطفيليات البالغة على الحركة وإنخفاض جوهري ملحوظ بنسبة (80%) في إطلاق الميكرو فيلاريا (MF) من الديدان الإناث في المختبر. وقد ظهرتخبط في الحركة تقريبا على نصف الحالات المصابة ب *L3* , المعالجة في المختبر وقد كانت حيوية استمرار حياة و تطور اليرقانات المصابة ب L3 والمعالجة ب siRNA التي تم فحصها في التجويف البريتوني للجرذان منخفضا بنسبة 45% في استكمال الديدان البالغة طبقا لما تم ملاحظته.

الخلاصة أن : طرحت النتائج بوضوح أن ipgm ضروري لكل من اليرقانات و المراحل البالغة من طفيل ب.ملاوي و أنها تلعب دور هام في تخلق الديدان الإناث. ولذا فإن النتائج تؤكد أن BM-iPGM عقار مرجح مستهدف مضاد للفلاريا .

Translated from English version into Arabic by Mohamed Gaafar, through

TRANSLATORS
WITHOUT BORDERS

马来丝虫不依赖于辅因子的磷酸甘油酸变位酶(iPGM)的体外基因沉默

Prashant Kumar Singh, Susheela Kushwaha, Mohd. Shahab, Manisha Pathak, and Shailja Misra-Bhattacharya

摘要

引言：在糖酵解/糖异生途径中，磷酸甘油酸变位酶催化由3-磷酸甘油酸和2-磷酸甘油酸之间的可逆转化，它存在于大多数细胞生物体中。磷酸甘油酸变位酶可分为依赖于辅因子的磷酸甘油酸变位酶(dPGM)和不依赖于辅因子的磷酸甘油酸变位酶(iPGM)两种类型。脊椎动物、酵母和许多细菌仅含有dPGM，而高等植物、线虫、古细菌和许多其他细菌仅含iPGM。少数细菌，包括大肠埃希菌和特定的古细菌和原生动物含有两种类型的磷酸甘油酸变位酶。在秀丽隐杆线虫中沉默*ipgm*的研究证实，该酶对该种线虫的生存性非常重要。因此，它可作为潜在的驱虫药物的靶标。在本研究中，应用RNA干扰（RNAi）技术研究马来丝虫ipgm在其生存力、微丝蚴释放和胚胎发生中的作用，以及对感染期幼虫进行后基因沉默。

结果：以siRNA体外*ipgm*基因沉默导致雌虫子宫内发育期的微丝蚴严重表型畸形，成虫游动性剧减约90%，在体外导致雌虫释放微丝蚴数量显著降低80%。体外处理的感染期3期幼虫近一半表现出动作迟缓。在沙鼠感染模型中研究siRNA处理的感染期3期幼虫的生存和发育情况，观察到成虫发育率减少约45%。

结论：结果清楚地表明，iPGM对于马来丝虫的幼虫和成虫阶段都起着重要作用，对于雌虫的胚胎发育是必不可少的。证实Bm-iPGM可作为一种推崇的抗丝虫的药物靶标。

Translated from English version into Chinese by Yang Pin, through

CHINESE CENTER FOR DISEASE CONTROL AND PREVENTION
NATIONAL INSTITUTE FOR PARASITIC DISEASES

Le silençage génique *in vitro* de l'enzyme phospoglycérate mutase indépendante (PGMi) chez la filaire parasite *Brugia malayi*

Prashant Kumar Singh, Susheela Kushwaha, Mohd. Shahab, Manisha Pathak, et Shailja Misra-Bhattacharya

Résumé

Contexte : L'enzyme phosphoglycérate mutase (PGM) catalyse l'interconversion du 2 et 3-phosphoglycérate (2- et 3-PG) dans les voies glycolytique et néoglucogénique qui sont présentes chez la majorité des organismes cellulaires. Elle est connue, soit sous la forme PGM co-facteur dépendante (PGMd), soit PGM co-facteur indépendante (PGMi). Les vertébrés, les levures, et de nombreuses bactéries ne présentent que la PGMd, alors que chez les végétaux supérieurs, les nématodes, les archées, et de nombreuses autres bactéries, seule la PGMi est présente. Un petit nombre de bactéries, y compris l'*Escherichia coli* et certaines archées, ainsi que certains protozoaires, possèdent les deux. Le silençage de la pgmi chez *Caenorhabditis elegans* (*C. elegans*) a démontré l'importance de cette enzyme dans la viabilité du parasite et, dès lors, son potentiel comme cible anthelminthique. Dans cette étude, le rôle de la pgmi chez *Brugia malayi* (*B. malayi*) dans la viabilité du parasite, l'émission microfilaire, l'embryogenèse, et le développement *in vivo* de la larve infectante après silençage génique ont été explorés en appliquant des études d'interférence par acide ribonucléique (ARN).

Résultats : Le silençage *in vitro* du gène *pgmi* par petits ARN interférents (pARNi ou siRNA) a entraîné de sévères malformations phénotypiques dans les stades de développement intra-utérins des vers femelles avec une réduction drastique (~90 %) de la motilité des parasites adultes et une réduction significative (80 %) de l'émission de microfilaires (mf) par les vers femelles *in vitro*. Près de la moitié des L3 infestantes traitées *in vitro* montraient un mouvement fortement ralenti. La survie *in vivo* et le développement des larves infestantes (L3) traitées par pARNi ont été étudiés dans la cavité péritonéale de mériones où une diminution d'établissement de vers à l'état adulte de ~45 % a été observée.

Conclusion : Les résultats indiquent clairement que l'enzyme PGMi est essentielle tant pour le stade larvaire que l'état adulte du parasite B. malayi et qu'elle joue un rôle clé dans l'embryogenèse des vers femelles. Les résultats valident donc l'hypothèse que l'enzyme Bm-PGMi représente une cible thérapeutique potentielle chez les filaires.

Translated from English version into French by Yves Goethals, through

TRANSLATORS
WITHOUT BORDERS

Сайленсинг гена независимой фосфоглицератмутазы (iPGM) филярии Brugia malayi in vitro

Прашант Кумар Сингх, Сушила Кушваха, Мохд. Шахаб, Маниша Патак и Шайлия Мизра-Бхаттачарья

Краткое изложение

История вопроса: ферменты фосфоглицератмутазы (PGM) катализируют взаимопревращение 2- и 3-фосфоглицерата в гликолитических/глюконеогенных путях, присутствующих в большинстве клеточных организмов. Они могут быть классифицированы как кофактор-зависимые PGM (dPGM) или кофактор-независимые PGM (iPGM). Позвоночные, дрожжевые грибы и многие бактерии обладают только кофактор-зависимыми фосфоглицератмутазами, а высшие растения, нематоды, археи и другие бактерии – только кофактор-независимыми. Небольшое количество бактерий, в том числе *Escherichia coli* и определенные археи и простейшие организмы, содержат оба вида фосфоглицератмутаз. Сайленсинг iPGM в *Caenorhabditis elegans* (*C. elegans*) продемонстрировал важное значение этого фермента для жизнеспособности паразитов, а следовательно, его потенциальное использование в качестве объекта антигельминтных препаратов. В данном исследовании роль фосфоглицератмутазы *Brugia malayi* (*B. malayi*) в жизнеспособности паразитов, откладывании микрофилярий, эмбриогенезе и развитии инвазионных личинок *in vivo* после сайленсинга генов изучалось путем применения исследований интерференции рибонуклеиновой кислоты (РНК).

Результаты: Сайленсинг гена фосфоглицератмутазы *in vitro* с помощью малой интерферирующей РНК вызывает глубокие фенотипические деформации во внутриутробных стадиях развития самок червей с радикальным снижением подвижности взрослых особей паразитов (~90%) и значительным сниженным отложением микрофилярий самками червей (80%) *in vitro*. Почти половина обработанных *in vitro* инвазионных личинок третьей стадии демонстрировали замедленное движение. Выживаемость и развитие *in vivo* обработанных малой интерферирующей РНК инвазионных личинок третьей стадии исследовалось в брюшной полости песчанок, где было обнаружено снижение распространения взрослых особей червя в ~45%.

Заключение: Полученные результаты явно указывают на то, что iPGM является жизненно важной как для личинок, так и для взрослых особей паразита *B. malayi*, и что она играет ключевую роль в эмбриогенезе самок червя. Таким образом, результаты подтверждают, что Bm-iPGM является предполагаемым объектом антифилярийных лекарственных средств.

Translated from English version into Russian by Elena McDonnell, through

TRANSLATORS
WITHOUT BORDERS

Silenciamiento genético *in vitro* del fosfoglicerato mutasa independiente (iPGM) en el parásito filarial *Brugia malayi*

Prashant Kumar Singh, Susheela Kushwaha, Mohd. Shahab, Manisha Pathak y Shailja Misra-Bhattacharya

Extracto

Reseña: La enzima fosfoglicerato mutasa (PGM, por sus siglas en inglés) cataliza la interconversión del 2-fosfoglicerato y del 3-fosfoglicerato en las vías glucolítica/gluconeogénica, presentes en la mayoría de los organismos celulares. Puede clasificarse en PGM dependiente cofactor (dPGM) o PGM independiente cofactor (iPGM). Mientras los vertebrados, las levaduras y muchas bacterias sólo poseen dPGM, las plantas, los nematodos, las arqueas y muchas otras bacterias sólo tienen iPGM, aunque una cantidad menor de bacterias, incluyendo la *Escherichia coli* y ciertos protozoos y arqueas, contiene ambas formas. El silenciamiento del *ipgm* en el *Caenorhabditis elegans (C. elegans)* ha demostrado la importancia de esta enzima en la viabilidad parasitaria y, por lo tanto, su potencial como diana de medicamentos antihelmínticos. En este estudio, se exploró el rol del ipgm del *Brugia malayi* (*B. malayi*) en la viabilidad parasitaria, en la liberación de microfilarias, en la embriogénesis y en el desarrollo *in vitro* de larvas infecciosas luego del silenciamiento genético mediante la aplicación de estudios de interferencia del ácido ribonucleico (ARN).

Resultados: El silenciamiento genético *in vitro* del *ipgm* por medio del ARN interferente pequeño (siRNA) conlleva graves deformidades fenotípicas en las etapas de desarrollo intrauterino de gusanos hembra, produciéndose una drástica reducción (~90%) en la motilidad de los parásitos adultos y una disminución importante (80%) en la liberación de microfilarias (mf) en gusanos hembra *in vitro*. Cerca de la mitad de las L3 infecciosas tratadas *in vitro* demostraron un movimiento muy lento. Se investigó la supervivencia y el desarrollo *in vivo* de las larvas infecciosas (L3) tratadas mediante siRNA en la cavidad peritoneal de jerbos, dónde se observó una reducción de, aproximadamente, un 45% en el establecimiento de parásitos adultos.

Conclusión: Los descubrimientos sugirieron claramente que el iPGM es esencial tanto para las etapas de larva como para las etapas adultas del parásito *B. malayi*, además de que desempeña un papel crucial en la embriogénesis de parásitas. Así, los resultados validan el Bm-iPGM como diana de medicamento putativo antifilarial.

Translated from English version into Spanish by David Acuña, through

TRANSLATORS
WITHOUT BORDERS
